# Cross-Cultural Adaptation and Validation of the Academic Resilience Scale-30 for Responses to Adverse Workplace Performance Evaluation Among Greek Public Healthcare Employees

**DOI:** 10.3390/healthcare14183050

**Published:** 2026-09-17

**Authors:** Maria Kapritsou, Vasiliki Papanikolaou, Maniadakis Nikos, Theodoros N. Sergentanis

**Affiliations:** 1 Oncological Sector, Hellenic Anticancer Institute ‘’Saint Savvas’’ Hospital of Athens, 11522 Athens, Greece; 2 Department of Public Health Policy, University of West Attica, 11521 Athens, Greece; bpapanikolaou@uniwa.gr (V.P.); nmaniadakis@uniwa.gr (M.N.); tsergentanis@uniwa.gr (T.N.S.)

**Keywords:** resilience, healthcare workforce, scale adaptation, psychometrics, ARS-30, Greece, public healthcare

## Abstract

**Background:** The Academic Resilience Scale-30 (ARS-30) was originally developed to assess cognitive, emotional, and behavioral responses following negative academic evaluation. Because structured performance appraisal has recently become an established component of the Greek public healthcare system, the present study examined whether this evaluative response framework could be meaningfully adapted to occupational performance evaluation. **Methods:** Test–retest reliability and descriptive analyses were conducted in an initial sample of 140 public healthcare employees, while EFA, internal-consistency analyses, and CFA were conducted in a separate larger sample of 483 employees. **Results:** Test–retest reliability was very high to excellent across items (ICC = 0.904–1.000). Internal consistency was acceptable for the total score (α = 0.799), good for Performance-Oriented Coping (α = 0.889), and acceptable for Negative Emotional Reactions/Passive Coping (α = 0.767). Positive Reappraisal/Growth Orientation (α = 0.705) and Acute Negative Emotional Reaction (α = 0.707) demonstrated marginally acceptable internal consistency and should therefore be interpreted cautiously. Item 23 was reverse-coded based on its semantic and conceptual direction. Exploratory factor analysis identified a preliminary four-component solution explaining 42.17% of the variance. However, the rotated solution included cross-loadings, and confirmatory factor analysis did not provide adequate support for the prespecified four-factor structure (CFI = 0.661, TLI = 0.629, RMSEA = 0.105, and SRMR = 0.105). **Conclusions:** Although some reliability findings were encouraging, the hypothesized four-factor structure was not supported by CFA and cannot currently be considered structurally validated. Further psychometric evaluation and model development are therefore required.

## 1. Introduction

Resilience is increasingly recognized as an important psychological resource for employees working in demanding healthcare environments. Public healthcare employees are often exposed to high workload, emotional pressure, organizational constraints, limited resources, and continuous changes in administrative and performance-related expectations. These conditions may challenge employees’ ability to maintain psychological well-being, adapt to workplace adversity, and sustain effective professional functioning over time [1,2,3].

In healthcare settings, resilience has been associated with the capacity to cope with stress, recover from adversity, maintain motivation, and continue functioning effectively despite difficult working conditions. Although resilience is not a fixed personal trait, it reflects a dynamic process involving cognitive, emotional, and behavioral responses to challenge [4]. This is particularly relevant in public healthcare systems, where employees must often manage both direct care-related demands and broader organizational pressures [1,2].

Recent international evidence has demonstrated that resilience is associated with a broad range of individual and organizational outcomes among healthcare professionals. Higher resilience has consistently been linked with lower levels of occupational stress and burnout, better psychological wellbeing, greater work engagement, improved job satisfaction, and enhanced professional functioning [1,2,5,6]. Furthermore, resilience has been associated with improved adaptation to organizational change, greater workforce retention, and higher quality of patient care [2,3,7]. Systematic reviews have highlighted resilience as a key protective resource supporting healthcare professionals working under prolonged occupational demands and organizational pressure [1,2,5]. Consequently, accurate assessment of resilience has become increasingly important for both research and workforce development within healthcare systems [8,9,10].

The Greek public healthcare sector represents a context in which resilience may be especially important. Public healthcare employees have experienced prolonged organizational pressure, increased accountability demands, and ongoing administrative reforms, including performance evaluation and goal-setting processes. Such conditions require employees not only to perform their duties but also to adapt to feedback, manage uncertainty, and remain engaged despite institutional and workload-related difficulties [7,8]. For this reason, the assessment of resilience in this population may provide useful information for workforce support, organizational development, and future intervention planning [1,2,10].

Despite the growing interest in resilience among healthcare workers, the measurement of resilience remains complex. Existing resilience instruments have often been developed for general, clinical, educational, or student populations and may not fully capture the specific challenges faced by public healthcare employees [8,9,10]. Several resilience instruments have been developed for clinical, community, and occupational populations, including measures that conceptualize resilience primarily as a relatively stable personal characteristic [10,11,12,13]. This response-oriented conceptual framework was considered particularly appropriate for the Greek public healthcare environment, where formal performance evaluation has become an established component of organizational practice. Unlike many resilience instruments that conceptualize resilience primarily as a stable personal trait, the ARS-30 evaluates individuals’ cognitive, emotional, and behavioral responses following formal negative evaluation. This response-oriented perspective is particularly relevant in organizational settings where employees are increasingly exposed to structured performance appraisal systems. Accordingly, the rationale for the present adaptation was not to transfer an academic resilience construct into healthcare practice, but to examine whether adaptive responses to evaluative adversity can be meaningfully assessed within a workplace performance evaluation context. Consequently, the ARS-30 was selected because it assesses how employees respond to evaluative workplace adversity rather than measuring resilience solely as a stable personality attribute [12].

When a scale is transferred to a new language, culture, or occupational context, linguistic translation alone is not sufficient. Cross-cultural adaptation requires careful consideration of conceptual equivalence, contextual relevance, and psychometric performance in the target population [14,15,16]. In contrast, the ARS-30 was specifically designed to evaluate cognitive, emotional, and behavioral responses following a negative evaluative event. Because formal performance evaluation has become an established component of the Greek public healthcare system, we considered this response-oriented framework particularly relevant for examining how healthcare employees react to adverse evaluative situations within their occupational environment.

The Academic Resilience Scale-30 (ARS-30), developed by Cassidy, is a multidimensional self-report instrument originally designed to assess students’ responses to academic adversity [12]. The scale focuses on how individuals respond cognitively, emotionally, and behaviorally to a hypothetical adverse situation. Although originally developed for academic settings, the underlying response patterns assessed by the ARS-30—such as coping, emotional reaction, persistence, and positive reappraisal—may also be relevant to occupational adversity when appropriately adapted to the workplace context [9,10,11,12].

The present adaptation does not assume that academic resilience and occupational resilience represent identical constructs [9,10,11,12]. The theoretical assumption underlying this adaptation is that formal performance evaluation, regardless of whether it occurs in educational or occupational environments, represents a structured evaluative challenge requiring similar cognitive, emotional, and behavioral regulatory processes. Rather, it is based on the theoretical premise that both academic and workplace settings require individuals to respond cognitively, emotionally, and behaviorally to adverse evaluative situations. Although the contextual sources of adversity differ, the underlying adaptive response processes may be comparable. Nevertheless, transferring an instrument from an academic to an occupational context requires empirical psychometric evaluation to determine whether these underlying response patterns are adequately represented within the target population [14,15,16,17].

In the original validation study, the ARS-30 demonstrated satisfactory psychometric properties. Exploratory factor analysis supported a three-factor structure consisting of Perseverance, Reflecting and Adaptive Help-Seeking, and Negative Affect and Emotional Response, explaining 42.4% of the total variance. The original scale showed high internal consistency for the global score (Cronbach’s α = 0.90), while the three subscales also demonstrated acceptable reliability, with Cronbach’s alpha values ranging from 0.78 to 0.83. Evidence of validity was also reported, including a significant positive association between ARS-30 global scores and academic self-efficacy, as well as support for discriminant validity through differences in responses to alternative academic adversity vignettes [12].

In the present study, the ARS-30 was translated into Greek and contextually adapted for use among public healthcare employees. The objective was to preserve the theoretical framework, item content, and response processes of the original ARS-30 while improving contextual relevance for the occupational setting. The adapted version, hereafter referred to as the ARS-30-PHE, represents a contextual adaptation of the original Academic Resilience Scale-30 (ARS-30) for use among public healthcare employees while preserving the theoretical framework and original item content of the parent instrument. The adaptation aimed to preserve the conceptual basis of the original instrument while making the scenario and item wording meaningful for employees working within the Greek public healthcare sector [13,14,15,16].

Given that the ARS-30-PHE represents an adaptation of an academic resilience measure to a new occupational and cultural context, empirical evaluation of its psychometric properties is required before it can be used confidently in research or practice [14,15,16,17]. In particular, it is necessary to examine its reliability, temporal stability, internal structure, and the extent to which its factor structure is supported in the target population [17,18].

No Greek adaptation of the ARS-30 for public healthcare employees has previously been reported. Moreover, no previous occupational adaptation of the ARS-30 was identified in the literature. Consequently, the present study addresses an important gap by providing the first adaptation and preliminary psychometric evaluation of the ARS-30 for Greek public healthcare employees.

Therefore, the purpose of the study was not only to translate the ARS-30 but also to examine whether its evaluative response framework remains psychometrically meaningful after transfer from academic assessment to public-sector workplace performance appraisal. Following the initial test–retest evaluation, additional psychometric analyses were conducted in a larger sample recruited from different public healthcare institutions to provide a more extensive evaluation of the instrument’s measurement properties. Because exploratory and confirmatory structural analyses were conducted in the same larger sample, the CFA should not be interpreted as independent structural cross-validation.

## 2. Methods

### 2.1. Study Design and Setting

This was a cross-sectional psychometric validation study conducted in the Greek public healthcare sector. Data were collected between May 2023 to September 2024 from employees working in public healthcare organizations and units of the national health system. The study focused on the evaluation of a Greek adaptation of the 30-item resilience scale originally developed by Cassidy as the Academic Resilience Scale (ARS-30) [12], adapted here to the context of public service and performance evaluation in the health sector.

### 2.2. Participants and Sampling

For the validation of psychometric instruments, commonly recommended guidelines suggest recruiting approximately 5 participants per item, with an absolute minimum of 3 participants per item, depending on item complexity and communalities [19,20,21]. Given that the ARS-30 consists of 30 items, an a priori target sample size of approximately 150 participants was set. A total of 140 employees ultimately completed the questionnaire. Eligible participants were (a) adults (≥18 years old), (b) currently employed in the Greek public sector within health-related services, and (c) able to read and understand Greek. Exclusion criteria were temporary staff without a stable employment relationship and individuals on long-term leave during the data collection period.

The study sample consisted predominantly of female nurses employed in the Greek public healthcare sector, with smaller representation from other healthcare professions. Sociodemographic characteristics are presented in Table 1. Basic sociodemographic data were collected, including age, gender, education, professional role, and years of work experience in the public sector.

Participation was voluntary and without financial or other incentives. Questionnaires were coded without direct personal identifiers; for participants included in the test–retest assessment, individual study codes were used solely to enable linkage between the two administrations. The questionnaire was distributed in paper form, and in selected services electronically, with identical content and instructions across modes. Convenience sampling was employed. Participants were recruited from three public healthcare institutions following approval from the respective hospital administrations. Recruitment was coordinated through the participating institutions, and all eligible employees were invited to participate voluntarily. This recruitment procedure was adopted to minimize potential investigator-related selection bias by avoiding direct participant selection by the research team. For the assessment of test–retest reliability, each participant was assigned an individual study code that enabled linkage of the first and second administrations without the use of direct personal identifiers. The questionnaire was re-administered after a 15-day interval using the same study code for record matching. Participants had been informed in advance about the planned second administration. All 140 participants who completed the first administration also completed the retest; therefore, no participants were lost to follow-up between T1 and T2. Participants were not provided with their previous responses by the research team at the second administration.

The total number of eligible employees who received or had access to the initial study invitation was not systematically recorded; therefore, a formal response rate could not be calculated for the initial sample.

A larger sample of 483 public healthcare employees was subsequently recruited from three public hospitals using convenience sampling. These hospitals were different from the institutions from which the initial sample of 140 participants was recruited; therefore, the two samples represented separate recruitment pools with no participant overlap. Invitations were distributed through the participating hospital administrations to eligible employees. The total number of employees who received or had access to the invitation was not systematically recorded; consequently, a formal response rate could not be calculated for this sample. This sample was used for exploratory factor analysis, internal-consistency analyses, and confirmatory factor analysis.

### 2.3. Instrument

The instrument used in this study was a Greek adaptation of the Academic Resilience Scale-30 (ARS-30), originally developed by Cassidy [12]. The original ARS-30 is a 30-item multidimensional self-report scale designed to assess cognitive, emotional, and behavioral responses to academic adversity. Respondents are presented with a hypothetical adverse academic scenario and are then asked to rate 30 statements describing possible reactions to that situation on a 5-point Likert-type scale.

For the purposes of the present study, the ARS-30 was translated into Greek and culturally adapted for use among public healthcare employees, with permission from the original developers. The adapted version was named the Academic Resilience Scale-30 for Public Healthcare Employees (ARS-30-PHE). The aim of the adaptation was to assess resilience-related responses to occupational adversity, particularly in the context of performance evaluation, organizational demands, and public-sector healthcare work.

To enhance contextual relevance, the original academic vignette was modified to reflect a workplace evaluation scenario. Specifically, participants were asked to imagine receiving a lower-than-expected performance evaluation, accompanied by critical but constructive feedback regarding their work performance. The term “tutor” in the original ARS-30 was replaced by “evaluator” to reflect the public-sector performance evaluation context. The item wording was adapted from academic challenges to work-related challenges, while preserving the conceptual meaning of the original items as closely as possible. The contextual adaptation was intentionally conservative. The principal modifications consisted of replacing the introductory academic vignette with a workplace performance evaluation scenario and replacing the term “tutor” with “evaluator”. Apart from these limited contextual modifications, the original wording of the questionnaire items, response format, and theoretical framework were preserved.

Items are rated on a five-point Likert scale ranging from 1 (strongly disagree) to 5 (strongly agree). Higher scores on adaptive items indicate stronger resilience-related responses, whereas higher scores on negative emotional response items indicate greater emotional vulnerability. Therefore, reverse scoring was applied where required to ensure interpretive consistency. In the present analysis, Item 23 was reverse-coded because its wording represented the opposite conceptual direction of the Acute Negative Emotional Reaction construct. This scoring direction was determined on the basis of the semantic and conceptual direction of the item relative to the negative emotional-response construct and was not derived from its observed exploratory factor loading.

The translation and cross-cultural adaptation process followed internationally recognized recommendations for the cross-cultural adaptation of self-report measures, consistent with the guidance proposed by Beaton et al. and the more recent practical recommendations described by Cruchinho et al. [14,15,16]. Two independent bilingual translators conducted forward translations from English into Greek. A synthesized Greek version was then produced by the research team. Two different bilingual translators, blinded to the original version, performed back-translation into English. The back-translated version was compared with the original ARS-30, and discrepancies were reviewed by an expert panel consisting of three senior nurses with extensive experience in the Greek public healthcare sector and employee performance evaluation. The panel was selected primarily to provide content and contextual expertise regarding the target occupational setting and evaluated semantic equivalence, conceptual relevance, contextual appropriateness, and clarity of the adapted workplace scenario and item wording. Minor wording refinements were introduced following the panel’s recommendations while preserving the conceptual content of the original ARS-30. The panel did not include a dedicated specialist in psychometrics, occupational/organizational psychology, or cross-cultural measurement methodology; accordingly, its role was limited to content and contextual review, while the measurement properties of the adapted instrument were subsequently evaluated empirically.

A small-scale pilot pretest was conducted with ten public healthcare nurses who were not included in either analytic sample. The purpose of this phase was to identify potential problems in comprehension, wording, acceptability, and contextual relevance before full administration rather than to conduct a psychometric evaluation. Participants reviewed the adapted workplace-evaluation scenario and questionnaire items and provided feedback regarding whether the wording was clear and understandable, whether the intended meaning of the items was apparent, and whether the scenario and terminology were appropriate to their public healthcare work context. The research team reviewed this feedback to identify wording requiring clarification. No major comprehension or interpretation problems were identified, and only minor wording refinements were introduced before final administration. This pretest was intended to assess comprehensibility and contextual appropriateness and did not constitute formal cognitive interviewing.

In the present study, the prespecified four-factor item configuration was subjected to empirical psychometric evaluation. Subscale scores were calculated according to this prespecified item allocation rather than by reassigning items post hoc on the basis of their strongest exploratory factor loadings. The EFA was used to evaluate the extent to which the observed item-loading pattern supported this proposed configuration. Items demonstrating weaker or discrepant psychometric performance were retained at this stage to preserve the content of the adapted 30-item instrument and to avoid post hoc item deletion or reassignment based on the same dataset used for structural evaluation. Their performance was instead reported explicitly to inform subsequent model-development and independent validation studies.

### 2.4. Data Collection

Data collection took place in selected hospitals and public healthcare units. After receiving permission from the respective management bodies, employees were informed about the study aims, voluntary participation, confidentiality, and the use of coded questionnaires without direct personal identifiers. Those who agreed to participate provided written informed consent.

Participants completed the questionnaire in approximately 10–15 min during or immediately after their working shift, depending on local organizational arrangements. All completed questionnaires were coded without personal identifiers. Paper questionnaires were stored securely and accessible only to the research team.

### 2.5. Statistical Analysis

Test–retest reliability and descriptive analyses were conducted using the initial sample of 140 public healthcare employees. Exploratory factor analysis (EFA/PAF), internal consistency reliability analyses (Cronbach’s α and McDonald’s ω), and confirmatory factor analysis (CFA) were conducted using the larger sample of 483 public healthcare employees. No missing data were identified in the final analytic dataset; therefore, no imputation procedures were required. Descriptive statistics were calculated for all sociodemographic variables and scale scores. Categorical variables were presented as absolute and relative frequencies, while continuous variables were summarized using means, standard deviations, minimum values, and maximum values.

Test–retest reliability was evaluated using the Intraclass Correlation Coefficient (ICC), calculated with a two-way mixed-effects, single-measure, consistency model (ICC(3,1)). To complement the item-level ICC estimates for the five-point ordinal responses, linearly and quadratically weighted Cohen’s kappa, exact agreement, and adjacent agreement were calculated for each item. Exact agreement was defined as an identical response at T1 and T2, whereas adjacent agreement was defined as a difference in no more than one response category. ICC values were interpreted as indicators of temporal stability, with higher values reflecting greater temporal consistency over the retest interval.

Internal consistency reliability was assessed using Cronbach’s alpha and McDonald’s omega for each prespecified subscale and for the directionally consistent total resilience score. Values of α ≥ 0.70 were used as a reference criterion for acceptable internal consistency, while values of α ≥ 0.80 were considered good [22]. Item 23 was reverse-coded before calculating F4 and the total resilience score because its wording represented the opposite conceptual direction of the Acute Negative Emotional Reaction construct.

The total resilience score was calculated in a directionally consistent manner so that higher values consistently reflected higher resilience. Specifically, the adaptive dimensions, Performance-Oriented Coping/Self-Regulated Learning and Positive Reappraisal/Growth Orientation, were combined with reverse-scored negative emotional response dimensions, Negative Emotional Reactions/Passive Coping and Acute Negative Emotional Reaction. ARS17 remained part of the 30-item ARS-30-PHE questionnaire and was retained in item-level screening, total-score reliability analysis, and EFA. Because ARS17 was not assigned to a factor in the prespecified four-factor configuration, it was excluded only from the CFA and other model-based analyses requiring explicit factor assignment. Thus, its exclusion from the CFA should not be interpreted as removal of ARS17 from the adapted instrument.

Construct validity was examined using exploratory factor analysis (EFA) and confirmatory factor analysis (CFA). Prior to EFA, sampling adequacy was evaluated using the Kaiser–Meyer–Olkin (KMO) statistic and Bartlett’s test of sphericity. Exploratory structural analyses were performed using Principal Axis Factoring (PAF), with Direct Oblimin and Promax oblique rotations, because correlations among the latent dimensions were expected. Pattern matrices, structure matrices, communalities, cross-loadings, and factor correlations were examined. Factor loadings of |0.30| or greater were considered salient. Items were considered structurally problematic when they demonstrated low target loadings, substantial non-target loadings, low communalities, inconsistency with the prespecified factor allocation, or problematic cross-loading. A problematic cross-loading was defined as the presence of two salient loadings differing in absolute magnitude by less than 0.20. Internal consistency was evaluated using Cronbach’s alpha and McDonald’s omega, while construct validity was further assessed using Composite Reliability, Average Variance Extracted, Maximum Shared Variance, Average Shared Variance, HTMT ratios, and the Fornell–Larcker criterion [23,24]. Corrected item–total correlations and Cronbach’s α and McDonald’s ω if item deleted were additionally examined as descriptive item-level reliability diagnostics. Local model fit was additionally evaluated by inspection of normalized and observed-minus-implied residuals and modification indices. These diagnostics were used to characterize sources of model misfit and were not used to modify the prespecified model post hoc.

Exploratory factor analysis (EFA/PAF) was conducted in the sample of 483 public healthcare employees to examine the latent structure of the adapted instrument. The proposed four-factor model was subsequently evaluated using confirmatory factor analysis (CFA) in the same sample. Model fit was evaluated using the chi-square statistic, Comparative Fit Index (CFI), Tucker–Lewis Index (TLI), Root Mean Square Error of Approximation (RMSEA), and Standardized Root Mean Square Residual (SRMR). Conventional thresholds of CFI and TLI ≥ 0.90 and RMSEA and SRMR ≤ 0.08 were used as reference criteria for acceptable model fit [10,11,25].

Statistical analyses were conducted using IBM SPSS Statistics for Windows, Version 25.0 (IBM Corp., Armonk, NY, USA).

Confirmatory factor analysis was performed using IBM SPSS AMOS version 25 with normal-theory Maximum Likelihood (ML) estimation. Because the ARS-30-PHE items use five-point ordinal response categories, item distributions were additionally examined for skewness and kurtosis when considering the assumptions of ML estimation.

### 2.6. Ethical Considerations

The study was conducted in accordance with the principles of the Declaration of Helsinki and the ethical standards of health research. Ethical approval was obtained from the Ethical Committee of West Attica University of Athens, Greece prior to data collection (ID: 18-04-2022/41892). All participants were informed about the aims, procedures, voluntary nature of participation, and their right to withdraw without consequences. Written informed consent was obtained from each participant. Study data were coded without direct personal identifiers and were treated confidentially; individual study codes were used where necessary to link the two test–retest administrations.

## 3. Results

### 3.1. Demographic Characteristics

A total of 140 public healthcare employees were included in the final analytic sample. The majority of participants were female (78.6%), aged between 31 and 40 years (37.1%), and employed as nurses (81.4%). Most participants did not hold a position of responsibility in the workplace (81.4%). Regarding years of work experience, the largest group reported 0–4 years of experience (38.6%), followed by 10–20 years (26.4%) and 5–9 years (22.9%). Detailed demographic characteristics are presented in Table 1. The mean total resilience score was 65.81 (SD = 12.86), with scores ranging from 33 to 90.

### 3.2. Τest–Retest Reliability

Test–retest reliability was examined using the 30 ARS-30-PHE items administered to the same 140 participants at two time points separated by a 15-day interval. Intraclass correlation coefficients indicated very high to excellent temporal stability across all items. ICC values ranged from 0.904 to 1.000, with the corresponding 95% confidence intervals indicating high precision of the temporal stability estimates (Table 2). Several items demonstrated perfect agreement (ICC = 1.000). Although these findings indicate excellent temporal stability, such estimates should be interpreted cautiously because the relatively short retest interval, potential memory effects, and limited within-subject variability between administrations may also have contributed to these exceptionally high coefficients. The lowest ICC values were observed for Items 14 and 15, although both remained within the range of very high agreement (Table 2). Test–retest reliability was also evaluated for the four exploratory subscales and the directionally consistent total resilience score. All construct-level ICC values demonstrated excellent temporal stability (F1 = 0.995, F2 = 0.993, F3 = 1.000, F4 = 0.990, Total = 0.996). Complementary ordinal item-level analyses were consistent with the high ICC estimates. Linearly weighted Cohen’s kappa values ranged from 0.901 to 1.000, while quadratically weighted kappa values ranged from 0.902 to 1.000. Exact agreement across individual items ranged from 91.43% to 100.00%, and adjacent agreement ranged from 96.43% to 100.00%. Across all 4200 paired item responses, 4135 responses (98.45%) were identical at T1 and T2, while 4184 responses (99.62%) were identical or differed by no more than one response category. Sixteen of the thirty items demonstrated 100% exact agreement.

### 3.3. Internal Consistency Reliability

Internal consistency was evaluated using Cronbach’s alpha and McDonald’s omega. Performance-Oriented Coping/Self-Regulated Learning demonstrated good internal consistency (α = 0.889; ω = 0.889), while Negative Emotional Reactions/Passive Coping demonstrated acceptable internal consistency (α = 0.767; ω = 0.772). Positive Reappraisal/Growth Orientation (α = 0.705; ω = 0.712) and Acute Negative Emotional Reaction (α = 0.707; ω = 0.723) demonstrated marginally acceptable internal consistency and should therefore be interpreted cautiously. For the 30-item total score, Cronbach’s α was 0.799, whereas the corresponding one-factor McDonald’s ω was lower (ω = 0.665), providing limited support for interpretation of the total item set as a single common dimension. Reliability results are presented in Table 3.

Corrected item–total correlations and item-deletion diagnostics were also examined. At the prespecified factor level, all corrected item–total correlations were positive and ≥0.306. The lowest values were observed for ARS1 within Factor 2 (r = 0.320), ARS10 within Factor 3 (r = 0.335), and ARS23 within Factor 4 (r = 0.306). Deletion of individual items produced only minor changes in subscale reliability: Cronbach’s α increased from 0.767 to 0.773 following deletion of ARS1 from Factor 2, from 0.705 to 0.706 following deletion of ARS10 from Factor 3, and from 0.707 to 0.715 following deletion of ARS23 from Factor 4. For the 30-item total score, 12 corrected item–total correlations were below 0.30 and ARS23 showed a negative corrected item–total correlation (r = −0.127). These item-deletion statistics were used only as descriptive diagnostics and no item was removed on their basis.

### 3.4. Factorial Validity

#### Exploratory Factor Analysis

Exploratory structural analyses were performed using Principal Axis Factoring (PAF). Sampling adequacy was good (KMO = 0.837), and Bartlett’s test of sphericity was highly significant (χ^2^(435) = 6265.755, *p* < 0.001), indicating that the correlation matrix was suitable for latent factor analysis. Factor retention was evaluated using Parallel Analysis, the original Velicer Minimum Average Partial (MAP) criterion, the revised MAP criterion, eigenvalues, scree plot inspection, and theoretical interpretability. Collectively, these factor-retention procedures did not provide convergent empirical support for the prespecified four-factor structure. These findings were treated as diagnostic evidence regarding the proposed measurement configuration and were not used to derive an alternative scoring structure.

A prespecified four-factor Principal Axis Factoring solution was examined in accordance with the measurement model under evaluation. The solution explained 42.17% of the total variance.

Both Direct Oblimin and Promax rotations were examined and yielded a broadly consistent pattern of structural complexity rather than a clean simple structure. Across the two rotations, twelve items (ARS1, ARS2, ARS4, ARS5, ARS7, ARS9, ARS12, ARS15, ARS17, ARS19, ARS20, and ARS23) were identified as problematic in at least one rotation because of low target loadings, substantial cross-loadings, low communalities, disagreement with the prespecified factor allocation, or lack of assignment within the proposed model. In particular, ARS2 and ARS4 loaded more strongly outside their proposed factor, ARS15 loaded more strongly on Factor 1 than on its proposed Factor 2, ARS7, ARS12, ARS19, ARS20 and ARS23 demonstrated substantial cross-loadings, whereas ARS17 showed very low communality (0.145) and remained unassigned in the prespecified four-factor model. Thus, 12 of the 30 items (40%) demonstrated at least one structural concern, indicating substantial instability in the proposed four-factor configuration rather than isolated item-level imperfections.

Taken together, these findings do not provide strong empirical support for the proposed four-factor scoring structure and indicate substantial structural instability at the item level (Table 4). The Direct Oblimin factor correlations were generally low, ranging in absolute magnitude from 0.058 to 0.345. The strongest association was observed between Factors 1 and 3 (r = 0.345), followed by Factors 2 and 4 (r = 0.200). The remaining correlations were small (F1–F2 = −0.058, F1–F4 = −0.065, F2–F3 = −0.072, and F3–F4 = 0.072).

### 3.5. Confirmatory Factor Analysis

The larger psychometric sample comprised 483 public healthcare employees. Most participants were female (*n* = 381, 78.9%) and nurses (*n* = 376, 77.8%); the largest age group was 31–40 years (*n* = 138, 28.6%), and 112 participants (23.2%) held a position of responsibility.

Prior to CFA, item skewness ranged from −1.334 to 1.147 and excess kurtosis ranged from −1.165 to 1.110. Shapiro–Wilk tests were significant for all items (*p* < 0.001), indicating departure from exact univariate normality; however, the magnitude of skewness and kurtosis was not extreme. The prespecified four-factor model was evaluated using confirmatory factor analysis. The model demonstrated poor fit according to all prespecified conventional fit criteria and therefore was not supported by the CFA. Accordingly, the proposed four-factor structure cannot be considered structurally validated in the present sample (Table 5).

### 3.6. Factor Correlations

The prespecified four-factor model demonstrated inadequate fit in the larger psychometric sample: χ^2^(371) = 2331.778, *p* < 0.001, CFI = 0.661, TLI = 0.629, RMSEA = 0.105, and SRMR = 0.105 (Table 5). The CFA converged without inadmissible solutions; all estimated residual variances were positive and the implied covariance matrix was positive definite. Standardized factor loadings ranged from 0.310 to 0.765. The lowest standardized loadings were observed for ARS1 (0.310), ARS10 (0.312), and ARS23 (0.345), while the remaining indicators ranged from 0.451 to 0.765. Standard errors were obtained for all freely estimated target loadings, and no improper parameter estimates were identified. Local-fit diagnostics were consistent with the poor global model fit. Among the 406 unique off-diagonal normalized residuals, 133 exceeded |1.96| and 82 exceeded |2.58|, with a maximum absolute normalized residual of 9.979. In addition, 107 observed-minus-implied correlation residuals exceeded |0.10|, and the maximum absolute correlation residual was 0.455. Modification-index diagnostics also indicated substantial localized model strain: of 493 fixed candidate parameters examined, 210 had modification indices greater than 3.84 and 96 exceeded 10. The largest modification indices involved the ARS28–ARS29 residual covariance (MI = 101.080), the ARS6–ARS14 residual covariance (MI = 82.227), and a cross-loading of ARS23 on Factor 1 (MI = 73.160). These diagnostics were examined to characterize model misfit; no parameters were freed and the prespecified model was not modified post hoc.

### 3.7. Construct Validity

Composite reliability ranged from 0.701 to 0.895 across the four factors. However, Average Variance Extracted (AVE) ranged from 0.333 to 0.417 and remained below the conventional 0.50 criterion for all factors, indicating insufficient convergent validity. Maximum Shared Variance (MSV) exceeded AVE for all four factors, while Average Shared Variance (ASV) ranged from 0.143 to 0.160. Heterotrait–Monotrait (HTMT) ratios ranged from 0.255 to 0.685 and remained below the conventional 0.85 threshold. In contrast, the Fornell–Larcker criterion was not satisfied for the Factor 1–Factor 3 and Factor 2–Factor 4 comparisons. Accordingly, convergent validity was not established, while evidence regarding discriminant validity was mixed and cannot be considered fully established, particularly given the poor fit of the underlying CFA model.

## 4. Discussion

The present findings should also be interpreted within the broader conceptual challenge of transferring an evaluative response instrument from education to occupational performance appraisal. Although the contextual source of adversity differs, both settings involve formal performance evaluation, external judgment, feedback processing, emotional regulation, and subsequent behavioral adaptation. The objective of the present study was therefore not to demonstrate that academic resilience and occupational resilience are identical constructs, but to examine whether adaptive responses to evaluative adversity remain measurable after contextual transfer. The failure of the prespecified four-factor structure to receive empirical support may therefore reflect meaningful contextual differences in the organization of resilience-related responses to adverse evaluation across academic and workplace settings. Although the present findings cannot establish construct non-equivalence as the sole explanation for the observed model misfit, they indicate that the response structure identified in an academic context should not be assumed to transfer unchanged to workplace performance evaluation.

The present study should be interpreted as a preliminary psychometric evaluation combining findings from an initial test–retest sample and a separate larger psychometric sample recruited from different public healthcare institutions. The inclusion of the larger sample allowed a more extensive psychometric evaluation of the adapted instrument; however, the exploratory and confirmatory structural analyses conducted in this sample should not be interpreted as independent structural validation [1,2,25].

A major strength of the ARS-30-PHE was its high temporal stability. Test–retest reliability was very high to excellent across all items, with ICC values ranging from 0.904 to 1.000. However, several items demonstrated perfect agreement (ICC = 1.000). These findings should be interpreted cautiously, as the relatively short retest interval and limited response variability between administrations may have contributed to these exceptionally high estimates. This suggests that participants’ responses remained highly stable over the retest interval. In the context of public healthcare work, where resilience-related responses may be influenced by ongoing organizational pressures, the observed temporal stability is an important finding. This temporal consistency indicates stability of responses over the retest interval but should not be interpreted as evidence that the instrument validly measures the broader construct of occupational resilience [26].

Internal consistency findings were more differentiated. The Performance-Oriented Coping/Self-Regulated Learning subscale demonstrated good internal consistency, while the Negative Emotional Reactions/Passive Coping subscale showed acceptable reliability. These findings indicate comparatively stronger internal consistency for these two prespecified item groups in the present sample, without establishing their structural validity as distinct latent constructs. In contrast, the Positive Reappraisal/Growth Orientation and Acute Negative Emotional Reaction subscales showed marginally acceptable internal consistency. These lower alpha values may be partly explained by the small number of items included in these factors, the exploratory nature of the factor solution, and the contextual adaptation of a scale originally developed for academic adversity [22,27]. Therefore, these subscales should not be interpreted as fully established independent dimensions at this stage. In addition to the relatively small number of items, the observed cross-loadings and the lack of support for the hypothesized four-factor structure may also have contributed to the lower internal consistency estimates. Consequently, further psychometric evaluation is required before these subscales can be considered stable independent dimensions.

The scoring procedure also highlighted an important psychometric issue. Item 23 was reverse-coded before calculating the Acute Negative Emotional Reaction factor and the directionally consistent total resilience score because its wording reflected the opposite conceptual direction of the negative emotional reaction construct. Τhe scoring decision was based primarily on item wording and conceptual direction. In translated and contextually adapted instruments, scoring assumptions should not be transferred automatically from the original version without empirical verification in the target population [14,15,16,26].

The present findings also suggest that transferring an instrument originally developed for academic adversity to an occupational setting may involve more than contextual adaptation alone. Although the wording modifications were intentionally conservative and the underlying theoretical framework was retained, workplace adversity may differ conceptually from academic adversity in important ways. Occupational resilience is influenced by organizational structures, professional responsibilities, interpersonal relationships, and workplace culture, all of which may affect how individuals respond to adverse evaluative situations. These contextual differences may partly explain why the prespecified factor structure was not supported in the present study despite preservation of the theoretical framework of the original instrument [28,29].

Although the directionally consistent total score demonstrated a Cronbach’s α of 0.799, internal consistency alone does not establish that the items represent a single interpretable latent construct. Additional reliability analysis yielded a lower one-factor McDonald’s ω of 0.665, providing limited support for a unidimensional interpretation of the global score. Accordingly, the total score should not presently be regarded as a validated or preferred global measure, and its use should remain exploratory pending further evaluation of the common variance underlying the item set.

The revised exploratory analyses provided a more rigorous evaluation of the latent structure of the Greek ARS-30-PHE. In contrast to the original analysis, the revised evaluation used Principal Axis Factoring with oblique rotation. The prespecified four-factor solution explained 42.17% of the total variance but did not produce a clean simple structure. Several items demonstrated low target loadings, substantial cross-loadings, disagreement with the proposed factor allocation, or low communalities. In particular, ARS2, ARS4, and ARS15 loaded more strongly outside their proposed factors, whereas ARS7, ARS12, ARS19, ARS20, and ARS23 demonstrated meaningful cross-loadings. ARS17 remained unassigned within the prespecified measurement model and showed the lowest communality (h^2^ = 0.145), indicating poor representation by the extracted factors. Taken together, the finding that 12 of the 30 items (40%) demonstrated structural problems indicates substantial instability in the proposed item–factor configuration. Accordingly, the exploratory findings do not provide strong empirical support for the proposed four-factor scoring structure, a conclusion that is further reinforced by the poor fit of the subsequent CFA.

Compared with the original ARS-30, the present findings demonstrate both similarities and important differences. Similar to the original instrument, the adapted version identified a multidimensional structure and demonstrated satisfactory internal consistency for the overall score. However, unlike the original validation, the present exploratory solution included several cross-loading items, two subscales demonstrated lower internal consistency, and the proposed measurement model was not supported by confirmatory factor analysis. These differences may reflect the challenges associated with transferring an instrument originally developed for academic adversity to a different occupational and cultural context, as well as differences in the characteristics of the target population [12].

The confirmatory factor analysis provided a more rigorous evaluation of the prespecified measurement model. The CFA provided clear evidence that the prespecified four-factor model did not adequately represent the observed data. All major fit indices failed to meet the stated criteria for acceptable model fit. Consequently, the hypothesized four-factor structure was not supported and cannot be considered structurally validated. This negative structural finding is informative for the present cross-context adaptation and indicates that the latent organization of responses to adverse workplace performance evaluation may differ from the proposed four-factor configuration [25]. One possible explanation is that workplace performance appraisal involves organizational, interpersonal, and professional factors that extend beyond the educational context in which the original instrument was developed.

The additional construct-validity analyses further reinforced the need for cautious interpretation. Although composite reliability was acceptable across the proposed factors, AVE remained below 0.50 for all four dimensions, indicating insufficient convergent validity. Evidence regarding discriminant validity was mixed: HTMT values remained within conventional limits, whereas the Fornell–Larcker criterion was not satisfied for two factor pairs and MSV exceeded AVE across all factors. These findings should be interpreted in conjunction with the poor CFA fit and therefore do not provide sufficient evidence to establish convergent or discriminant validity of the proposed four-factor measurement structure.

The factor correlations also provided meaningful information. A moderate positive association was observed between Performance-Oriented Coping and Positive Reappraisal/Growth Orientation, suggesting that adaptive behavioral and cognitive responses may be related aspects of resilience. Similarly, the stronger correlation between Negative Emotional Reactions/Passive Coping and Acute Negative Emotional Reaction indicates overlap between the two emotional vulnerability dimensions. This overlap may partly explain why the four-factor CFA model did not show adequate fit. It is possible that the negative emotional response items represent a broader common dimension rather than two clearly distinct factors in this population.

At its present stage of psychometric development, the ARS-30-PHE should be restricted to exploratory, group-level research aimed primarily at further evaluating resilience-related responses to adverse workplace performance evaluation. The present findings do not support its interpretation as an established measure for general research applications, individual assessment, or high-stakes organizational decision-making. Any use of the total or subscale scores should therefore remain exploratory pending further structural validation and independent replication.

### Study Limitations

Several limitations should be acknowledged. The CFA was conducted in a sample of 483 participants, which was not considered a limitation of the present confirmatory analysis. Rather, the poor model-fit indices indicate that the prespecified four-factor model did not adequately represent the observed covariance structure. Accordingly, the lack of structural support should be interpreted as evidence of model misspecification in the present sample rather than attributed to insufficient sample size. Further research should therefore focus on evaluating alternative theoretically plausible measurement models, including formal comparison with the original three-factor ARS-30 structure, and subsequently replicating the resulting structure in independent samples. In addition, the CFA was estimated using normal-theory Maximum Likelihood despite the five-category ordinal response format. Although item-level skewness and kurtosis were not extreme, the ordinal nature of the indicators and departures from exact normality should be considered when interpreting the CFA results. Future studies should examine the robustness of the findings using an ordinal estimator such as WLSMV or DWLS.

In addition, both samples were recruited using convenience sampling and were predominantly composed of nurses (81.4% in the initial sample and 77.8% in the larger psychometric sample), with women also representing the majority of participants. These sampling characteristics limit the generalizability of the findings to the broader population of public healthcare employees and particularly to professional groups that were underrepresented in the present study. Future validation studies should therefore recruit more professionally diverse and gender-balanced samples using broader sampling strategies. Second, although the larger psychometric sample included 483 participants, EFA and CFA were conducted in the same sample. Consequently, the confirmatory analysis does not constitute independent cross-validation of the exploratory structural findings, and replication in a separate sample remains necessary. Third, the study was conducted among public healthcare employees in Greece, and the findings may not generalize to other healthcare settings, private-sector healthcare employees, or other public-sector occupational groups. Fourth, the present study focused mainly on internal structure, reliability, and descriptive score characteristics. Additional evidence is needed regarding criterion-related validity, convergent validity with other resilience measures, and predictive validity in relation to work-related outcomes. Because the ARS-30 was originally developed for educational settings, it remains possible that certain response patterns are specific to academic evaluation and therefore may not fully generalize to occupational performance appraisal.

In addition, consistent with the vignette-based design of the original ARS-30, participants in the present study responded to a hypothetical adverse workplace performance evaluation and were not selected on the basis of having recently experienced negative performance feedback. Actual recent experience of adverse evaluation may influence the salience and organization of cognitive, emotional, and behavioral responses; therefore, the measurement structure may differ among employees who have recently experienced such feedback. Future studies should directly compare vignette-based responses with those obtained from employees following an actual recent adverse performance evaluation.

Furthermore, formal response rates could not be calculated because the total numbers of eligible employees who received or had access to the study invitations were not systematically recorded. Although convergent and discriminant validity were examined, the resulting evidence was insufficient to establish construct validity of the proposed factor structure. Criterion-related and predictive validity have not been established, and the ARS-30-PHE has not yet been validated against an established measure of occupational resilience. Future studies should therefore examine associations with theoretically relevant external measures and prospective occupational outcomes.

Although complementary weighted-kappa and agreement analyses corroborated the high temporal consistency estimates, the exceptionally high test–retest results should still be interpreted cautiously given the relatively short 15-day retest interval and the possibility of memory effects. Future studies using longer retest intervals and independent samples are warranted to further evaluate temporal stability.

The composition of the expert panel represents an additional limitation of the cross-cultural adaptation process. Although the panel members provided substantial clinical, organizational, and performance-evaluation experience within the target public healthcare context, the panel did not include dedicated expertise in psychometrics, occupational/organizational psychology, or cross-cultural measurement methodology. Future adaptation studies would benefit from multidisciplinary expert panels incorporating these complementary areas of expertise.

Future studies should include larger independent samples to further evaluate the internal structure of the ARS-30-PHE, examine measurement invariance across relevant demographic groups, establish convergent and discriminant validity using established resilience measures, evaluate criterion-related and predictive validity, and compare alternative theoretically plausible latent measurement models, including the original three-factor ARS-30 structure and models permitting cross-loadings, such as Exploratory Structural Equation Modeling (ESEM), followed by independent cross-validation.

## 5. Conclusions

The present study provides a preliminary psychometric evaluation of the adaptation of the ARS-30 evaluative response framework to public-sector workplace performance appraisal. Although some reliability findings were encouraging, the structural analyses did not support the prespecified four-factor measurement model. The findings therefore indicate that the latent organization of resilience-related responses observed in an academic context may not transfer cleanly to adverse workplace performance evaluation without further psychometric development.

The hypothesized four-factor structure was not supported by the CFA and therefore cannot be considered structurally validated. Although the exploratory analysis identified patterns corresponding to performance-oriented coping, negative emotional reactions, positive reappraisal, and acute negative emotional reaction, the proposed configuration did not adequately reproduce the observed covariance structure. Accordingly, the four subscale scores should not presently be interpreted as established latent dimensions. In addition, two subscales demonstrated marginal internal consistency. Therefore, the subscale scores should be considered preliminary and interpreted cautiously.

The present findings should be interpreted as preliminary psychometric evidence regarding the Greek adaptation of the ARS-30-PHE. Although the instrument demonstrated encouraging reliability characteristics, the available evidence does not yet support definitive structural validation or routine applied use. The present evidence should therefore not be interpreted as establishing the ARS-30-PHE as a validated measure of occupational resilience in the broad sense; rather, it provides preliminary psychometric evidence regarding the assessment of responses to adverse workplace performance evaluation.

Accordingly, the ARS-30-PHE should presently be considered appropriate only for exploratory, group-level research primarily directed toward further psychometric evaluation while further validation studies in independent samples continue to evaluate its measurement properties.

## Figures and Tables

**Table 1:** Demographic characteristics of participants, N = 140.

Variable	Category	N	%
**Gender**	Female	110	78.6
	Male	30	21.4
**Age group**	20–30	37	26.4
	31–40	52	37.1
	41–50	25	17.9
	51–60	25	17.9
	61–70	1	0.7
**Education**	Higher Technological Institute	53	37.9
	Higher University Degree	17	12.1
	MSc	37	26.4
	PhD	2	1.4
	Other	31	22.1
**Profession**	Nurse	114	81.4
	Other health professions	26	18.6
**Position of responsibility**	No	114	81.4
	Yes	26	18.6
**Years of work experience**	0–4 years	54	38.6
	5–9 years	32	22.9
	10–20 years	37	26.4
	21–30 years	11	7.9
	>31 years	6	4.3

**Table 2:** Test–retest reliability of ARS-30-PHE items, N = 140.

Item	ICC	95% CI
**1**	1.000	1.000–1.000
**2**	1.000	1.000–1.000
**3**	1.000	1.000–1.000
**4**	1.000	1.000–1.000
**5**	1.000	1.000–1.000
**6**	1.000	1.000–1.000
**7**	1.000	1.000–1.000
**8**	1.000	1.000–1.000
**9**	1.000	1.000–1.000
**10**	1.000	1.000–1.000
**11**	1.000	1.000–1.000
**12**	0.998	0.997–0.998
**13**	0.973	0.962–0.980
**14**	0.904	0.869–0.931
**15**	0.930	0.903–0.949
**16**	0.985	0.978–0.989
**17**	0.990	0.986–0.993
**18**	0.995	0.993–0.996
**19**	0.941	0.919–0.957
**20**	0.981	0.974–0.987
**21**	0.991	0.988–0.994
**22**	0.984	0.978–0.988
**23**	1.000	1.000–1.000
**24**	0.982	0.975–0.987
**25**	1.000	1.000–1.000
**26**	0.943	0.922–0.959
**27**	0.961	0.947–0.972
**28**	1.000	1.000–1.000
**29**	1.000	1.000–1.000
**30**	1.000	1.000–1.000

**Table 3:** Internal consistency reliability of the ARS-30-PHE, N = 483.

Scale/Subscale	Items	Cronbach’s α
**Performance-Oriented Coping/Self-Regulated Learning**	11, 13, 16, 18, 20, 21, 22, 24, 25, 26, 27, 30	0.889
**Negative Emotional Reactions/Passive Coping**	1, 3, 5, 15, 19, 28, 29	0.767
**Positive Reappraisal/Growth Orientation**	2, 4, 8, 9, 10	0.705
**Acute Negative Emotional Reaction**	6, 7, 12, 14, 23 *	0.707
**ARS1–ARS30**		0.799

* Item 23 was reverse-coded before calculating the Acute Negative Emotional Reaction subscale.

**Table 4:** Principal Axis Factoring (PAF) results with Oblimin rotation for the ARS-30-PHE (N = 483).

Item	Factor 1	Factor 2	Factor 3	Factor 4
**ARS1**	−0.268	0.295	0.257	0.045
**ARS2**	0.391	−0.239	0.271	0.367
**ARS3**	−0.180	0.518	0.159	0.083
**ARS4**	0.353	−0.077	0.250	0.312
**ARS5**	−0.060	0.430	0.091	0.221
**ARS6**	−0.140	−0.066	0.076	0.573
**ARS7**	−0.003	0.341	0.166	0.438
**ARS8**	0.200	−0.111	0.596	−0.010
**ARS9**	0.032	−0.288	0.540	0.067
**ARS10**	−0.016	0.139	0.615	−0.176
**ARS11**	0.548	−0.142	0.090	0.112
**ARS12**	0.089	0.334	−0.046	0.486
**ARS13**	0.592	−0.172	0.162	0.191
**ARS14**	0.022	0.094	−0.172	0.668
**ARS15**	−0.429	0.309	0.187	0.325
**ARS16**	0.683	0.057	0.054	0.004
**ARS17**	0.291	0.139	0.127	−0.101
**ARS18**	0.527	0.042	0.263	0.019
**ARS19**	0.049	0.523	0.061	0.321
**ARS20**	0.459	0.087	0.306	−0.107
**ARS21**	0.463	0.077	0.236	0.077
**ARS22**	0.633	0.095	0.124	−0.211
**ARS23**	−0.235	0.013	−0.304	0.460
**ARS24**	0.645	0.093	0.003	0.011
**ARS25**	0.712	0.017	0.020	−0.071
**ARS26**	0.655	0.095	−0.172	0.096
**ARS27**	0.766	−0.053	−0.142	0.008
**ARS28**	0.046	0.745	−0.083	0.073
**ARS29**	0.102	0.803	−0.063	−0.101
**ARS30**	0.661	0.100	0.015	−0.118

**Table 5:** Confirmatory factor analysis fit indices and factor correlations of the four-factor ARS-30-PHE model, N = 483. (**A**) Confirmatory factor analysis fit indices; (**B**) factor correlations.

(**A**)					
**Fit Index**	Observed Value	Conventional Criterion			
**χ^2^(df)**	2331.778 (371)	Non-significant preferred			
***p*-value**	<0.001	>0.05			
**CFI**	0.661	≥0.90			
**TLI**	0.629	≥0.90			
**RMSEA**	0.105	≤0.08			
**SRMR**	0.105	≤0.08			
(**B**)					
**Factor**		F1	F2	F3	F4
**F1—Performance-Oriented Coping/Self-Regulated Learning**		1.000	−0.081	0.662	−0.091
**F2—Negative Emotional Reactions/Passive Coping**		−0.081	1.000	−0.174	0.639
**F3—Positive Reappraisal/Growth Orientation**		0.662	−0.174	1.000	0.107
**F4—Acute Negative Emotional Reaction**		−0.091	0.639	0.107	1.000

## Data Availability

The data presented in this study are available from the corresponding author upon reasonable request. The data is not publicly available due to privacy and ethical restrictions.
